# A Facile Fabrication of Biodegradable and Biocompatible Cross-Linked Gelatin as Screen Printing Substrates

**DOI:** 10.3390/polym12051186

**Published:** 2020-05-22

**Authors:** Pei-Leun Kang, Yu-Hsin Lin, Kalpana Settu, Ching-Shu Yen, Chin-Yi Yeh, Jen-Tsai Liu, Ching-Jung Chen, Shwu-Jen Chang

**Affiliations:** 1 Cardiovascular Surgery, Department of Surgery, Kaohsiung Veterans General Hospital, Kaohsiung 81362, Taiwan; plkang@vghks.gov.tw (P.-L.K.); yhlin28@vghks.gov.tw (Y.-H.L.); 2 Department of Electrical Engineering, National Taipei University, New Taipei 23741, Taiwan; kalpana@gm.ntpu.edu.tw; 3 Department of Biomedical Engineering, I-Shou University, Kaohsiung 82445, Taiwan; eva1989201@hotmail.com (C.-S.Y.); isu10550003a@cloud.isu.edu.tw (C.-Y.Y.); 4 College of Materials Science and Opto-Electronic Technology, University of Chinese Academy of Sciences, Beijing 100049, China; 5 School of Opto-Electronic Technology, University of Chinese Academy of Sciences, Beijing 100049, China

**Keywords:** gelatin, crosslinking, flexible substrate, biocompatibility, screen-printed electrode

## Abstract

This study focuses on preparation and valuation of the biodegradable, native, and modified gelatin film as screen-printing substrates. Modified gelatin film was prepared by crosslinking with various crosslinking agents and the electrode array was designed by screen-printing. It was observed that the swelling ratio of C-2, crosslinked with glutaraldehyde and EDC/NHS (1-ethyl-3-(3-dimethylaminopropyl) carbodiimide/N-hydroxysuccinimide) was found to be lower (3.98%) than that of C-1 (crosslinked with only glutaraldehyde) (8.77%) and C-0 (without crosslinking) (28.15%). The obtained results indicate that the swelling ratios of both C-1 and C-2 were found to be lower than that of C-0 (control one without crosslinking). The Young’s modulus for C-1 and C-2 was found to be 8.55 ± 0.57 and 23.72 ± 2.04 kPa, respectively. Hence, it was conveyed that the mechanical strength of C-2 was found to be two times higher than that of C-l, suggesting that the mechanical strength was enhanced upon dual crosslinking in this study also. The adhesion study indicates that silver ink adhesion on the gelation surface is better than that of carbon ink. In addition, the electrical response of C-2 with a screen-printed electrode (SPE) was found to be the same as the commercial polycarbonate (PC) substrate. The result of MTT (3-(4,5-dimethylthiazol-2-yl)-2,5-diphenyl tetrazolium bromide) assay suggested that the silver SPE on C-2 was non-cytotoxic toward L929 fibroblast cells proliferation. The results indicated that C-2 gelatin is a promising material to act as a screen-printing substrate with excellent biodegradable and biocompatible properties.

## 1. Introduction

In order to study the electrical activity of biological cells, electrode arrays can provide useful information. In general, electrode arrays have been fabricated on hard substrates such as silicon [1], glass [2], and plastics [3]. However, the reliable communication between a biological cell and an electrode would be seriously affected by the mechanical mismatch between the soft biological tissues and the rigid electronic device [4,5]. Numerous studies have been carried out to construct electrode arrays on flexible substrates such as polyimide [6], parylene [7], and polydimethylsiloxane (PDMS) [8].

Non-toxic, lightweight, and relatively inert PDMS as an electroactive substrate possessing superior optical transparency has been commonly used as biosensor in biomedical, industrial, and environmental analyses [9,10,11]. Hence, in order to meet the requirements for specific applications in the biological field, several researchers significantly studied the surface modification of PDMS with bioactive molecules [12,13,14]. Wu et al. treated the PDMS surface by bioactive chlorogenic acid to modify the surface properties of PDMS [15]. The development of micro patterns of gelatin hydrogels on PDMS for the culture of induced pluripotent stem cell (iPSC)-derived cardiomyocytes was reported by Nawroth et al. [16]. However, surface treatment for improving cell adhesion and proliferation would be affected by various limiting factors such as the poor degradation and biocompatibility of the cell, the hydrophobic surface, and poor adhesion property of PDMS [17]. Therefore, the potential application of natural bioactive molecules such as gelatin and natural polymers was studied as an alternate direct substrate and the studies indicated that they could facilitate enhancing the degradation and biocompatibility of cells. 

The increased biocompatibility, biodegradability, non-toxic, and low-cost are the major advantages of gelation. Gelatin obtained by the thermal denaturation of collagen, possesses biodegradation with excellent biocompatibility, making it extensively used in the biomedical field [18,19,20,21,22]. Gelatin possesses outstanding properties for the cell–biomaterial interactions, including exposure of ligands, hydrophilicity, and surface roughness that promote cell attachment and proliferation [23,24]. In addition, gelation consists of various functional groups (OH, C=O, NH, and NH_2_) which facilitate the surface modification to improve its applicability in the field of biosensor and tissue engineering [25]. However, in some cases depending upon the fabrication process, gelatin substrates usually lack mechanical strength and high swelling behavior, which has prevented this unique biomedical material from being used as an electrode substrate. Therefore, there is a great need to develop a size stable gelatin substrate with enhanced mechanical strength for electrode substrate applications. Several attempts have succeeding in improving the mechanical strength of gelatin substrate and a significant improvement of the gelatin membrane strength was achieved after two-step crosslinking reactions [26]. The crosslinking method chosen to stabilize gelatin structures for biomedical applications is crucial. Glutaraldehyde has been widely used as a gelatin crosslinker and it provides good improvement in mechanical properties and scaffold stability [27,28]. The another most widely used chemical crosslinking method for gelatin relies on EDC/NHS (1-ethyl-3-(3-dimethyl aminopropyl) carbodiimide/ N-hydroxysuccinimide). The EDC/NHS crosslinking method has notable advantages, including a high conversion efficiency, mild reaction conditions, and excellent preservation of gelatin biocompatibility [23,29].

Among various fabrication techniques, the traditional method very often used to prepare the electrode for biosensor is known as screen-printing technology [30,31,32,33,34,35]. The screen-printed electrode is prepared by printing a pattern of choice onto the substrate followed by the solidification of printed pattern via either heating or UV irradiation. Thus, the screen-printing technique seemed to be simple and effective. The circuit pattern on the substrate could function as the electrical stimulation and could also measure the cellular growth behavior [36]. Among many types of screen-printing substrates, flexible substrates received greater attention in biomedical application [37,38]. 

The objective of this study is to discover the potentiality of natural polymers to act as substrates for screen-printing electrode arrays. Among the natural polymers, gelatin not only exhibited improved biodegradation but also possessed excellent biocompatibility and hence the film was selected as a substrate for screen-printing.

The main aim of this study is to explore the effects of various crosslinking agents on modifying gelatin film and using the crosslinked gelatin as a screen-printing substrate. Widely used sensing electrodes such as carbon and silver electrode arrays were screen-printed onto the crosslinked gelatin substrate, and basic parameters including swelling ratio, mechanical strength, and electrode adhesion test were evaluated. The cytotoxic effects of the crosslinked gelatin substrate and electrode arrays were investigated with L929 fibroblast cells using MTT (3-(4,5-dimethylthiazol-2-yl)-2,5-diphenyl tetrazolium bromide) assay. In this study, biodegradable gelatin film with enhanced size stability, lower swelling, and higher mechanical strength was successfully developed using dual crosslinking materials and evaluated as a novel screen-printing substrate.

## 2. Materials and Methods

### 2.1. Chemicals and Reagents

Type A gelatin, 1-ethyl-3-(3-dimethyl aminopropyl) carbodiimide (EDC), N-hydroxysuccinimide (NHS), and glutaraldehyde were purchased from Sigma (St. Louis, MO, USA). Polycarbonate (PC) substrate was obtained from Jan Yan Print Int’l Corp (Taoyuan City, Taiwan) and used as received without any modification. All the chemicals used in this study were of reagent grade.

### 2.2. Preparation of Gelatin Film

Gelatin was dissolved in deionized (DI) water at 60 °C to prepare 15 *w*/*v* % gelatin solution. The gelatin solution was poured into a Petri dish and then air-dried at room temperature for 24 h. Three types of gelatin samples were prepared in this study: (1) gelatin film without any crosslinking (C-0, no crosslinking); (2) gelatin film crosslinked with 2% glutaraldehyde (pH 4.8) for 24 h (C-1, single crosslinking); and (3) gelatin film crosslinked with 0.50% EDC/0.18% NHS (pH 6.4) for 24 h followed by crosslinking with 2% glutaraldehyde for 24 h (C-2, dual crosslinking). Finally, these resultant gelatin films were washed repeatedly with DI water to remove any traces of reacting agents and then air-dried in an oven at 40 °C overnight. All the prepared gelatin films were stored in a vacuum desiccator at room temperature. The morphology of the gelatin film was examined by a scanning electron microscope (SEM, Hitachi-4700, HORIBA, Kyoto, Japan). The gelatin film samples were sputter-coated with gold prior to SEM examination.

### 2.3. Swelling Test of Gelatin Film

In order to obtain the swelling film, the gelatin film was immersed into phosphate-buffered saline (PBS) at room temperature. At predetermined time intervals (1, 2, 3, 4, 5, 10, 20, 30, and 60 min), the film was removed from PBS and the film area was immediately measured (A_1_). The swelling ratio was calculated by using Equation (1) with A_0_ as the surface area of gelatin film before immersing in PBS. Each measurement experiment was repeated three times and expressed as average ±SD.

Swelling ratio = (A_1_ − A_0_)/A_0_ (1)

### 2.4. Mechanical Strength Test of Gelatin Film

The mechanical property tests were performed according to the ASTM D882 standard test method [39]. Gelatin films were cut into 1 cm × 6 cm rectangular shape and soaked in 0.1 M PBS (pH 7.4) for 24 h. The mechanical properties of these soaked gelatin films were calculated and recorded automatically by using a mechanical testing machine (Tinius Olsen, Horsham, PA, USA) at a crosshead speed of 10 mm/min. 

### 2.5. Fabrication of Screen-Printed Electrode (SPE) on Gelatin 

Carbon ink (SC-1010, ITK) and silver ink (NT-6307-2, PERM TOP) were printed onto the crosslinked gelatin and polycarbonate (PC) substrates by using a screen-printing machine (NSP-1A, Yulishih Industrial, New Taipei City 235, Taiwan) equipped with a 200 threads per inch polyester screen and polyurethane (PU). The size of all substrates was 1 × 3 cm^2^. The printed carbon-SPE and silver-SPE were dried at 60 °C for 30 min and 120 °C for 60 min, respectively.

### 2.6. Adhesion Test of the SPE

The adhesion strength of the screen-printed electrodes was evaluated by using a tape test according to ASTM D 3359-95 [40] to evaluate the effect of the carbon and silver ink adhesion to the crosslinked gelatin film substrate. The extent of adhesion between the inks and the substrate was analyzed by measuring the fraction of detached area after the test. The adhesion was evaluated by comparison with description and illustration in the ASTM D3359 manual. An evaluation scale (5B to 0B) was provided, where 5B indicates the best and 0B indicates the poorest.

### 2.7. Cyclic Voltammetry (CV) Measurement

The CV measurement was carried out using an IM6-eX electrochemical workstation (ZAHNER Zennium IM6, ZAHNER-elektrik GmbH & Co. KG, Kronach, Germany). The three-electrode system consisted of the screen-printed electrode as a working electrode, an Ag/AgCl wire as a reference electrode, and a platinum wire as a counter electrode. The CV scanning was performed at a scan rate of 100 mV/s with 0.1 mM, pH 7.2 potassium ferricyanide (K_3_Fe(CN)_6_) as the redox probe.

### 2.8. Cell Biocompatibility Assay

The biocompatibility test of gelatin film was performed according to ISO 10993 [41] by MTT assay using L929 fibroblast cells. MTT (3-(4,5-dimethylthiazol-2-yl)-2,5-diphenyl tetrazolium bromide) assay was used to evaluate the cell viability based on the mitochondrial conversion of the tetrazolium salt into a purple colored formazan product at an absorbance of 570 nm. The mouse fibroblast cell line L929 was cultured in Dulbecco’s modified Eagle medium (DMEM) supplemented with 10% fetal bovine serum (FBS), 100 U/mL penicillin, and 100 mg/mL streptomycin. Each sample was placed into one well in a 24-well plate and L929 cells were seeded on each well at 2 × 10^4^ cells/well. After 1, 2 or 3 days incubation, the original medium in each well was replaced with 100 μL MTT solution (5 mg/mL), and then the wells were incubated for 4 h at 37 °C in 5% CO_2_ incubator to enable the formation of formazan crystals. After removing the solution, dimethyl sulfoxide (DMSO) was added to all the wells and mixed thoroughly to dissolve the dark blue crystals. After a few minutes in order to ensure that all crystals were dissolved, the plates were read at 570 nm on a multi-well scanning ELISA reader (Thermo Scientific, Waltham, MA, USA).

### 2.9. Statistics

All the data were expressed as mean ± standard deviation (SD). The data were compared by one-way analysis of variance (ANOVA) to evaluate differences among the groups. A difference with *p* < 0.05 was considered statistically significant. 

## 3. Results and Discussion

### 3.1. Characterization of Gelatin Films 

#### 3.1.1. Morphology of Gelatin Film

**Figure 1:**
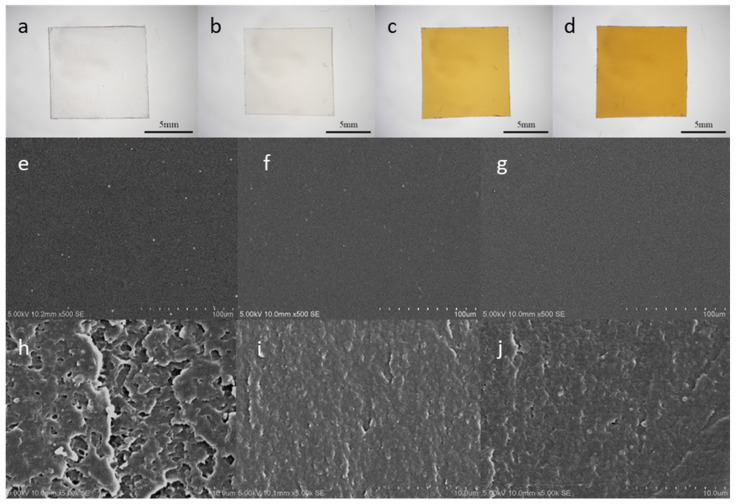
Optical photographs: (**a**) polycarbonate (PC), (**b**) C-0 (without crosslinking), (**c**) C-1 (crosslinked with only glutaraldehyde), (**d**) C-2 (crosslinked with EDC/NHS (1-ethyl-3-(3-dimethylaminopropyl) carbodiimide/N-hydroxysuccinimide) and glutaraldehyde); and scanning electron microscopy (SEM) images of surface: (**e**) C-0, (**f**) C-1, (**g**) C-2; and cross-section: (**h**) C-0, (**i**) C-1, (**j**) C-2.

Optical photographs and SEM images of PC, gelatin without any crosslinking (C-0), the single crosslinked gelatin film (C-1), and the dual crosslinked gelatin film (C-2) are presented in Figure 1. The optical photographs showed that the crosslinked gelatin films became yellow, suggesting the formation of a successful crosslinking structure. The SEM surface image showed that all gelatin films had a smooth surface, and moreover, the cross-section of C-1 and C-2 gelatin films showed a finer scale microstructure. This indicates that the crosslinking could effectively increase the compactness of the gelatin film [28], and such a smooth and compact gelatin surface is appropriate for screen-printing.

#### 3.1.2. Swelling Ratio of Gelatin Films

**Figure 2:**
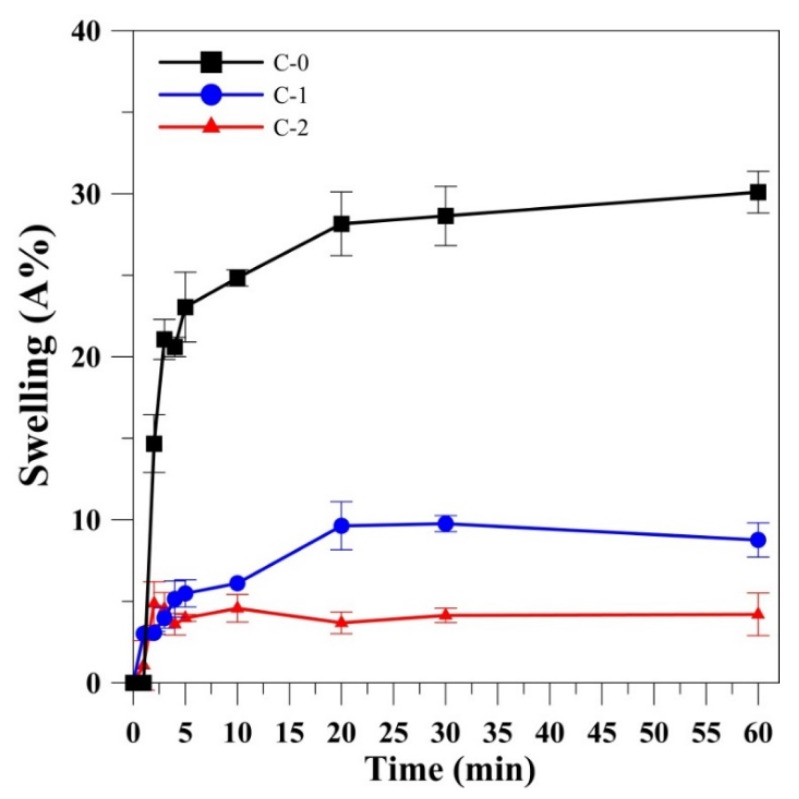
Swelling ratio of un-crosslinked (C-0), single crosslinked (C-1), and dual crosslinked (C-2) gelatin films in phosphate-buffered saline (PBS) at room temperature.

Figure 2 shows the swelling ratio measured at different time intervals for the C-0, C-1, and C-2 films. The swelling ratio for C-0 was increased drastically and reached saturation in 20 min with the swelling ratio of 28.15% and remained constant up to 60 min. Similarly, for C-1 the swelling ratio increased with time and attained 8.77% at 60 min, whereas for the dual-crosslinked gelatin film (C-2), the swelling ratio reached saturation in 5 min with the swelling ratio of 3.98% and remained constant up to 60 min, which is lower than C-0 and C-1. Gelation could adsorb water molecules as it is hydrophilic in nature. Upon incorporation of glutaraldehyde, the swelling property of the gelatin film was found to decrease possibly due to the increase in hydrophobicity of the matrix [42]. Another reason that could be attributed to this phenomenon was the increase in the crosslinking density between the glutaraldehyde and gelatin [43]. When glutaraldehyde was added to gelatin, the reaction between the amine (NH_2_) group of gelatin and the carbonyl (C=O) groups of glutaraldehyde would occur leading to the formation of a gelatin hydrogel network [27]. EDC/NHS crosslinking of gelatin film along with glutaraldehyde further reduced the swelling behavior of the gelatin film which could be possibly due to high crosslinking at longer duration (48 h). This is also possible from the production of short-range molecular crosslinks since reaction of EDC/NHS with gelatin matrices brought gelatin films more low-swelling structure [44]. In general, the degree of swelling was reduced for the polymer with high crosslinking and hence among all, the dual-crosslinked gelation film possessed a small rate of swelling indicating the low water adsorption capacity and increased hardness of the material [45].

#### 3.1.3. Mechanical Properties of Gelatin Films

**Figure 3:**
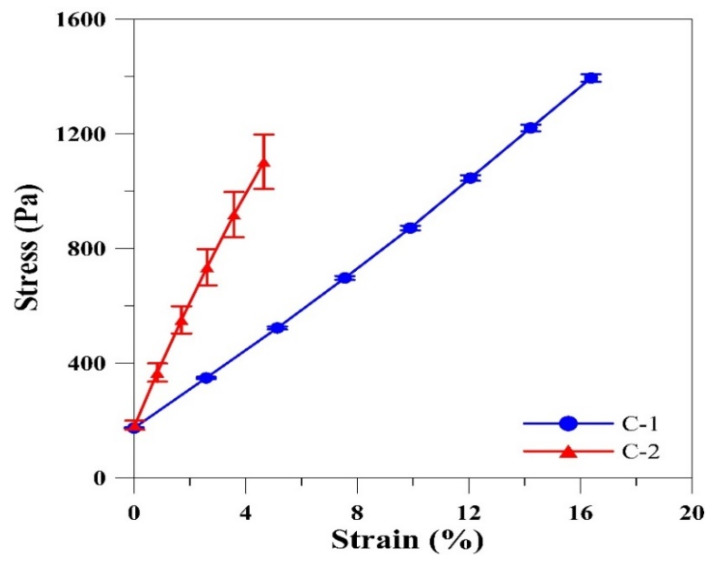
Stress–strain curves recorded from C-1 and C-2 gelatin films.

It was reported that glutaraldehyde crosslinking affects the stiffness of gelatin films [31]. Figure 3 shows the typical stress–strain curves recorded from gelatin films crosslinked with glutaraldehyde (C-1) and EDC/NHS/glutaraldehyde (C-2). A decrease in the extensibility and increase in the stress at break were observed for the C-2 gelatin film. The calculated Young’s modulus for the C-1 and C-2 gelatin films was 8.55 ± 0.57 and 23.72 ± 2.04 kPa, respectively. From the results, it was noted that an increase in the Young’s modulus would result in lower elasticity and higher size stability. This was possibly due to the compact space between the films contributed by higher crosslinking density. Thus, the structure of film was retained without any stretching. Cao et al. [32] reported a similar trend for polycarbonate film. This result indicates the improved mechanical strength of C-2 gelatin film and hence dual crosslinking makes gelatin film highly durable to physical pressure and is suitable for screen-printing. The mechanical strength test cannot be performed in the un-crosslinked gelatin film due to its poor mechanical properties. C-2 gelatin film was used as a substrate for printing electrode arrays.

### 3.2. Gelatin Film as Screen-Printing Electrode Substrate

Screen printing has evolved as a potential fabrication tool because it enables simple, rapid, and inexpensive electrode array preparation on a large scale [33]. In this work, we use C-2 gelatin film as a substrate on which carbon and silver electrode arrays were realized by employing screen-printing technique. 

#### 3.2.1. Adhesion Test of SPE 

**Figure 4:**
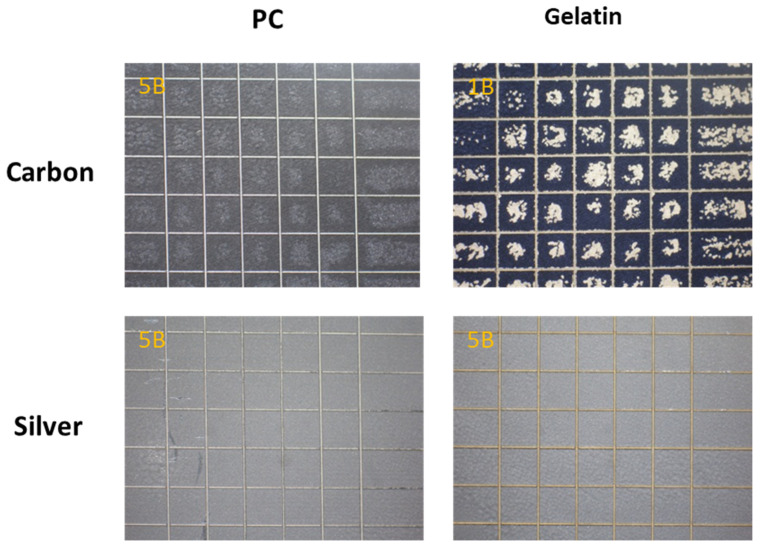
Optical microscopic images of adhesion test results for the screen-printed electrodes (SPes) on C-2 gelatin and PC substrates. Adhesion is assessed on a 0B to 5B scale, where 0B is the poorest and 5B is the best.

Adhesion strength is a significant factor for the reliability and functionality of metal electrode arrays onto various substrates. Both carbon and silver inks were screen-printed onto C-2 gelatin and PC substrates. In order to determine the adhesion capacity of the crosslinked C-2 gelatin film, the percentage of the adhesion was determined according to the procedure explained by ASTM D-3359-95 standard test methods and compared with PC substrate. From the test results (Figure 4), the screen-printed carbon ink and silver ink onto the PC film were rated as 4B and 5B, respectively. Carbon ink on C-2 gelatin film revealed poor adhesion (Grade-1B). However, silver ink on C-2 gelatin film exhibited strong adhesion (Grade-5B). The adhesion test confirmed that the silver electrode has a strong adhesion strength to the C-2 gelatin film substrate. Hence, silver screen-printed electrode was chosen for subsequent experimental analysis.

#### 3.2.2. Electrochemical Characterization of SPE

**Figure 5:**
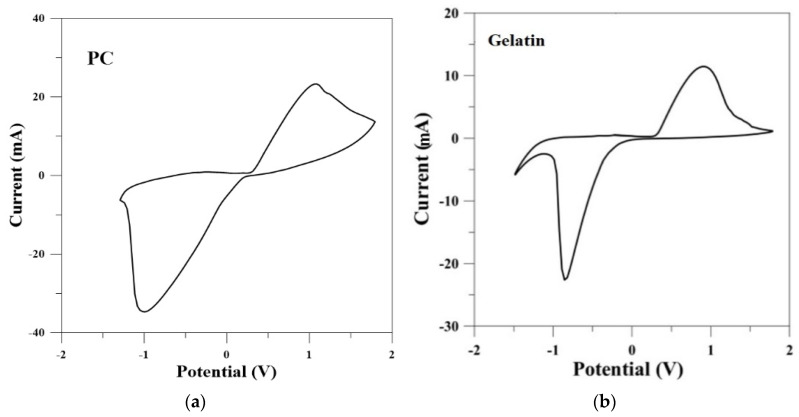
Cyclic voltammograms of silver SPEs on (**a**) PC and (**b**) C-2 gelatin substrates, measured in 0.1 mM K3[Fe(CN)6]. Scan rate 100 mV/s, reference electrode Ag/AgCl, counter electrode Pt.

The fabricated silver SPEs on C-2 gelatin film were characterized by cyclic voltammetry (CV) in potassium ferricyanide solutions and their performances compared with silver SPEs on PC substrate. Analytical data obtained from CV studies are shown in Figure 5. The results showed that the cyclic voltammograms for Ag electrode on PC and C-2 gelatin film almost exhibited the same common features. There were two redox peaks in each curve, which could be attributed to the redox of ferric ions. The upward peak is an anodic peak, reflecting the oxidation process from ferrous ion to ferric ion. Correspondingly, the downward peak is a cathodic peak, reflecting the reduction process from ferric ion to ferrous ion [34]. The sigmoidal response and its degree of symmetry indicated the irreversible nature (between silver ink and potassium ferricyanide) of the electroactive substances. This CV response suggests that the SPEs on soft gelatin substrate are very suitable for obtaining electrical signals from biological cells.

#### 3.2.3. Cell Viability Assay

**Figure 6:**
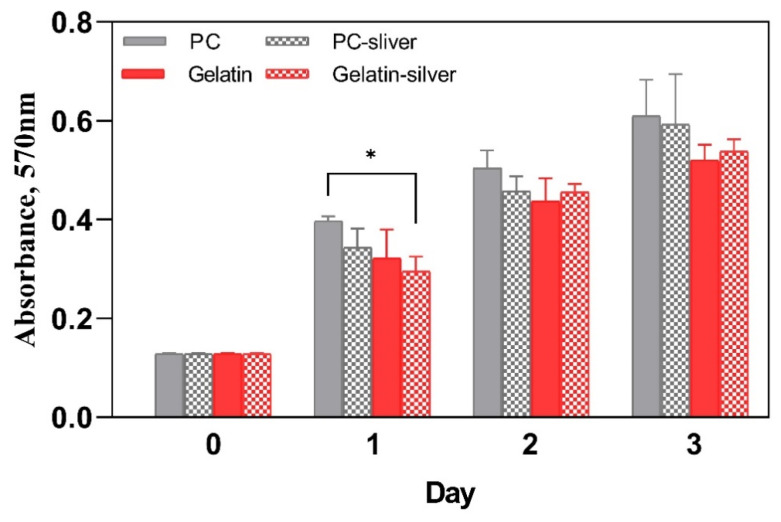
The biocompatibility test of gelatin film by MTT (3-(4,5-dimethylthiazol-2-yl)-2,5-diphenyl tetrazolium bromide) assay using L929 fibroblast cells. Data were expressed as means with standard deviations (mean ± SD). Statistical significance was set at a level of * *p* < 0.05 when compared with the control group.

MTT assay was executed to test the cell viability on C-2 gelatin substrate. L929 fibroblast cells were cultured on PC film and C-2 gelatin film for three days both in the presence and absence of Ag-SPE and the biocompatibility test with MTT assay results are shown in Figure 6. The MTT assay results exhibited that the proliferation of L929 fibroblasts is insignificant on C-2 gelatin substrate and C-2 gelatin substrate with Ag-SPE. On the first day, the cells proliferated, and then the growth became stagnant for all groups, although PC film and Ag-SPE gelatin film showed significant difference (*p* < 0.05). This result clearly indicates that C-2 gelatin substrate and Ag-SPE are not cytotoxic toward cell proliferation. Thus, the C-2 gelatin film could provide a biocompatible surface with exposed ligands that promotes cell attachment and proliferation by integrin-mediated interactions [23].

## 4. Conclusions

In this study, biodegradable gelatin film with enhanced size stability, lower swelling, and higher mechanical strength was successfully developed and evaluated as a screen-printing substrate. The results showed that the C-2 film facilitated effective screen-printing. Moreover, the swelling behavior of gelation film was not affected by the immersion of SPEs in PBS solution. Furthermore, the C-2 gelatin film with silver ink produced a harmless effect toward cells growth. Thus, gelatin-based electrode arrays with biocompatible characteristics could potentially be used as electronic devices for continuous real-time monitoring of human physiological signals. By applying both screen-printing and biopolymer crosslinking techniques, an inspired interdisciplinary platform could be developed for wearable or portable electronic devices in the field of biomedical engineering.
